# A comparative analysis of the effectiveness of laser correction with a mechanical and laser microkeratome for myopia

**DOI:** 10.5935/0004-2749.2023-0292

**Published:** 2024-09-16

**Authors:** Chaoge Liu

**Affiliations:** 1 Department of Clinical Medicine, Lugansk State Medical University, Luhansk, Ukraine

**Keywords:** Myopia, Lasers, Cornea, Keratomileusis, Laser in situ

## Abstract

**Purpose:**

Myopia, or nearsightedness, is one of the most common eye conditions worldwide. However, a comparison of the effectiveness of different laser-assisted interventions is lacking. Thus, we aimed to compare the efficacy and safety of LASIK and IntraLASIK in addressing myopia.

**Methods:**

The study was conducted in two ophthalmology clinics in Beijing, China, in 2022. A total of 84 patients (152 eyes) with different degrees of myopia were examined and underwent LASIK (n=46, 80 eyes) or IntraLASIK (n=38, 72 eyes). Keratometry, corneal topography, pachymetry, visual acuity evaluation, and corneal biomechanical analysis were performed before and after the intervention.

**Results:**

IntraLASIK produced more precise flaps than LASIK, with deviations of <8 mm and 0.1 mm from the intended thickness and diameter, respectively. LASIK resulted in nonuniform flaps, with thickness deviations of 5-86 mm. IntraLASIK demonstrated a superior efficacy for patients with severe myopia and thin corneas, with a mean spherical equivalent of 0.9 D at 6 months compared to the 1.4 D for LASIK. Approximately 91% and 83% of the patients with mild to moderate and severe myopia, respectively, achieved results within ± 0.49 D from the refractive target with IntraLASIK.

**Conclusions:**

Corneal hysteresis and corneal resistance factor decreased with an increase in laser intensity, and they decreased faster with thinner corneas. Thus, IntraLASIK is more useful than LASIK in patients with thin corneas and severe myopia.

## INTRODUCTION

Myopia affects 22%-36% of the population and poses significant challenges to eye health^(1)^. Traditional excimer laser procedures such as laser-assisted In situ keratomileusis (LASIK) have been the standard treatment for myopia correction. It involves creating corneal flaps with a microkeratome and reshaping the cornea with precision^(2^,^3)^. However, creating uniform flaps remains a challenge^(4)^. FemtoLASIK, a newer approach, utilizes femtosecond laser technology for precise flap creation, which enhances the outcomes^(5)^. ReLEx Smile (Carl Zeiss, Jena, Germany) employs high-precision lasers to create a lenticule within the corneal stroma, which improves functionality and ensures quicker recovery^(6)^. Despite these innovations, comparative studies on the different laser technologies for myopia correction are limited. In this study, we aimed to compare the efficacy and safety of LASIK and IntraLase-Assisted Laser In Situ Keratomileusis (IntraLASIK) in addressing myopia.

Comparative studies on the structural, functional, anatomical, and biomechanical properties of the cornea before and after refractive surgery are limited. Furthermore, the available studies either investigate the effectiveness of individual treatments or ignore the dynamic changes in these parameters^(3^,^7^,^8^,^9)^. We seek to narrow the gap with this study.

In this study, we aimed to determine the effect of IntraLASIK and LASIK technologies on corneal biome-chanics and eye refraction in patients with thin corneas. IntraLASIK and LASIK were selected because of their extensive utilization and recognition within medical practice. IntraLASIK is a contemporary and efficient approach that utilizes femtosecond laser technology for the creation of a corneal flap. This enables a more precise correction and enhances the predictability of outcomes. Conversely, LASIK is one of the most prevalent methods for myopia correction that has been extensively researched and is characterized by well-documented outcomes.

The objectives of the study are as follows: (1) to compare the response of corneal flaps to IntraLASIK and LASIK, (2) to compare the refractive outcomes of patients with thin corneas after IntraLASIK and LASIK, (3) to detect biomechanical changes in the thin corneas throughout the treatment processes, and (4) to analyze postoperative and intraoperative risks of IntraLASIK and LASIK.

## METHODS

### Participants

The study was conducted at two ophthalmology clinics in Beijing, China, in 2022. A total of 84 patients (152 eyes) were recruited and divided into two groups: LASIK intervention (n = 46, 80 eyes) and IntraLASIK intervention (n = 38, 72 eyes). The LASIK group included 26 female patients and 20 male patients who were aged 20-47 years (mean age, 31.3 ± 4.5 years). The Intra-LASIK group included 22 female patients and 16 male patients who were aged 22-45 years (mean age, 33.5 ± 5.1 years). The corneal thickness in both groups was <520 µm, with 60 patients (71%) exhibiting a corneal thickness of 470-500 µm.

### Study plan and structure

Patients with myopia and thin corneas were recruited from two ophthalmology clinics and randomly allocated into two groups, LASIK and IntraLASIK. LASIK and IntraLASIK were performed according to established protocols. Details of the surgical procedure, such as flap thickness and diameter, optical zone, and treatment area size, were recorded. The patients were followed up for 1 year after the surgery. The outcome measures were as follows: corneal parameters (keratometry, corneal topography, pachymetry, and visual acuity), biomicros-copic examination, corneal biomechanical characteristics (corneal hysteresis [CH], corneal resistance factor [CRF], and central corneal thickness [CCT]), spherical equivalent (SEQ), uncorrected visual acuity (UCVA), and best-corrected visual acuity (BCVA).

### Experimental procedures

All the participants underwent ophthalmological examinations before (baseline) and after the surgery. The postoperative examination time points were as follows: day 3, 6 months, and 1 year. Changes in the biometric characteristics and visual function were measured throughout the study period, and the results between the two groups were statistically compared.

### Procedures and equipment

All the patients underwent keratometry, corneal topography, pachymetry, and visual acuity assessment. Keratometry was used to measure the corneal curvature and assess its shape and dimensions. Corneal topography was used to map the corneal surface and identify any shape abnormalities. Pachymetry was used to determine the corneal thickness, assess its condition, and prepare for surgery. The CCT and peripheral corneal thickness (PCT) were measured at four arcuate zones (0-2, 2-5, 5-7, and 7-10 mm). The pachymetry data were obtained using Visante optical coherence tomography (OCT; Carl Zeiss Meditec, Jena, Germany). Visual acuity was evaluated to determine the degree of visual impairment. Additionally, biomicroscopic examination was conducted.

Corneal biomechanical parameters such as CH, CRF, and CCT were calculated using the ORA; Reichert Int., Depew, New York, USA). The percentage in CCT reduction from the baseline was also determined.

The patients were diagnosed by licensed ophthalmologists in the clinics where the study was conducted. The average time from diagnosis to seeking medical care was approximately 2 years.

IntraLASIK involved the following two steps: corneal flap creation with a 60 kHz IntraLase femtosecond laser (Abbot Medical Optics, California, USA) and laser ablation with a Microscan-2000 (MicroLabs, Kyiv, Ukraine). The IntraLASIK flap parameters were as follows: flap thickness, 100 µm; flap diameter, 9 mm; hinge angle, 45°; and side-cut angle, 70°. The pulse repetition rate of the laser system was 300 Hertz, and the spot size was 0.9 mm. The optical zone diameter ranged from 5.8 to 6.5 mm. The treatment zone diameter ranged from 8 to 8.5 mm, as specified in the tissue-sparing ablation protocol.

LASIK was performed in the standard manner using the Microscan-2000 (MicroLabs, Kyiv, Ukraine). The pulse repetition rate of the laser system was 300 Hertz, and the spot size was 0.9 mm. The corneal flap was created with the automatic M2 microkeratome (Moria S. A., Rue Georges Besse, France). Two cutting head sizes, 90 µm and 100 µm, were used. The expected flap diameter was 8.5-8.7 mm, and the flap pedicle was positioned at the 12 o’clock position. A temporal incision was used in the right eye, and a nasal incision was used in the left eye. The optical zone diameter ranged from 5.8 to 6.4 mm, and the treatment zone diameter ranged from 8 to 8.4 mm.

In LASIK, the optical zone size varied according to the preoperative corneal thickness and stage of myopic progression. The treatment zone size depended on the desired flap diameter. The vertical and horizontal flap dimensions and the pedicle width were measured intraoperatively.

Given the risk of flap immobility due to contact with the sensor, the corneal thickness on the third postoperative day was measured using the noncontact method. The flap surface and the corneal bed were also evaluated.

### Postdischarge examination

After being discharge from the hospital, the patients were examined in specially equipped ophthalmology clinics. The patients’ vision was evaluated, including the UCVA and BCVA. The CCT and PCT were measured using pachymetry and OCT. Biomicroscopy was conducted to assess the structure and contour of the corneal flap. The corneal biomechanical parameters, such as CH and CRF, were assessed using the ORA. Subsequently, the ophthalmologists discussed the results with the patients and offered further recommendations. The postdischarge examinations were conducted according to the study protocol, which allowed for the evaluation of long-term surgical outcomes and detection of any potential postdischarge complications.

### Inclusion and exclusion criteria

Patients diagnosed with spherical or mixed-form myopia that required correction and those who could consent to participating in the study were included in the study. The exclusion criteria were as follows: presence of contraindications to LASIK or IntraLASIK, presence of other ophthalmological or general medical conditions that could affect the study outcomes, women who were pregnant or breastfeeding at the time of vision correction, and failure to meet the image quality requirements during the preoperative examination.

### Surgical technique of tissue-sparing surgery

#### Preoperative preparation:

All patients underwent comprehensive ophthalmological examination, including refraction measurement, central corneal thickness (CCT), and anterior and posterior segment evaluation.

#### Operative technique:

The surgical intervention was performed using a femtosecond laser to create corneal flaps.

The flap was lifted, and an excimer laser was used to reshape the cornea according to the individual parameters of the patient.

Special attention was paid to minimizing tissue removal to preserve the structural integrity of the cornea.

### Ethical statement

This study was conducted in accordance with international norms and ethical principles (Declaration of Helsinki). Anonymity and confidentiality were guaranteed to the patient. The study was approved by the Ethics Committee of the Beijing Medical Institute (No: 399; | date |). All patients provided informed written consent.

### Statistical analysis

Data were analyzed using Statistics 10 (IBM SPSS Statistics, New York, USA). Data are presented as means and standard deviations. The preoperative and postoperative data within each group were compared using the two-tailed paired-sample Student’s t-test. This test was selected because the metrics of the same patients before and after the surgery were being assessed. The differences were considered significant at a p-value of ≤0.05.

## RESULTS

No complications were detected in the preoperative and postoperative periods.

The mean baseline CH was 8.5 ±1.1 mmHg (range, 6.4-12.0 mmHg). Approximately 50% of the corneas (80 eyes) had a CH of 8.0-10.0 mmHg. A lower CH (≤8 mmHg) was observed in 38 eyes, and a higher CH (≥10.0 mmHg) was observed in 34 eyes. The mean baseline CRF was 8.6 ± 1.4 mmHg (range, 7.4-12.6 mmHg). The mean baseline CCT of all the included eyes was 496.1 ± 18.3 µm.

Both groups had some degree of myopic degeneration at baseline. The mean SEQ ranged from 7.19 ± 2.34 D in the LASIK group to 7.44 ± 2.27 D in the IntraLASIK group. The UCVA was 0.037 ± 0.004 D in both groups, and the BCVA was 0.83 ± 0.16 D. The mean CCT was 495.4 ± 18.5 µm.

**Figure 1:**
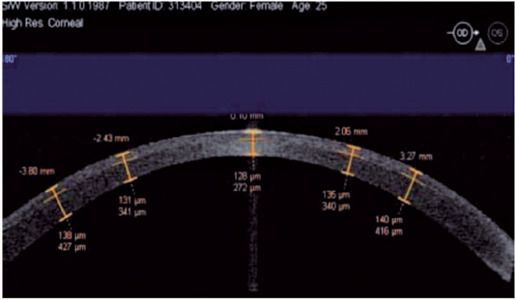
Corneal thickness measured 3 days after LASIK using optical computed tomography (magnifcation: 10 × 3). The orange lines represent the fap thickness.

The mean CCT obtained 3 days after LASIK was 126.9 ± 44.9 µm (intended, 100 µm; range, 80-187 µm). The mean CCT measured at 3 mm temporally (right eyes) and nasally (left eyes) was 140.9 ± 13.2 µm. The postoperative CCT in the LASIK group was 137.9 ± 19.9 µm. The PCT values varied across different zones of the eye because the flap had a meniscus shape, particularly at the periphery. The average flap size was 8.62 ± 0.3 mm, with a flap pedicle of 4.1 mm (Figure 1).

**Figure 2:**
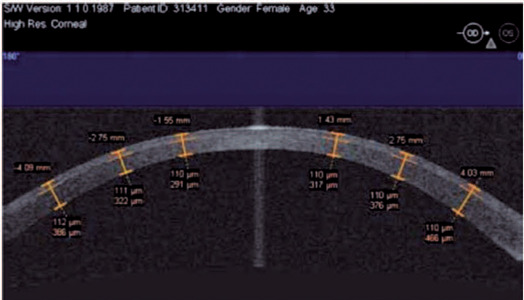
Corneal thickness measured 3 days after IntraLASIK using optical computed tomography (magnifcation: 10 × 3). The orange lines represent the fap thickness.

The planned and actual IntraLASIK flap thicknesses slightly differed by approximately 8 µm. The mean deviation from the intended flap diameter was 0.14 ± 0.06 mm. The excised flap was 9.2 ± 0.14 mm in diameter, with a pedicle width of 2.7 mm (Figure 2).

The contour of the flap edge was clear after IntraLASIK, but not after LASIK. No flap dislocations were detected in either group. However, there was a prominent response at the edge on the first postoperative day.

Preoperatively, the patients in both groups were divided into two smaller groups according to myopic severity. The first subgroup comprised patients with moderate to mild myopia, with a diopter range of -0.50 to -5.00. The second subgroup included patients with myopia of 6.25-10.00 D. After IntraLASIK, the average myopic regression at the 1-year follow-up in subgroup 1 was 0.46 ± 0.20 D (p≤0.01). Approximately 91% of the eyes examined were within ±0.49 D of the refractive target, and 97% were within ±1 D of the refractive target. In subgroup 2, IntraLASIK yielded similar outcomes, with a mean myopic regression of 0.95 ± 0.23 D (p≤0.01). Approximately 83% of the eyes were within ±0.5 D of the refractive target, and 83% were within ±1 D of the refractive target. The SEQs after IntraLASIK are detailed in table 1.

**Table 1:** Mean spherical equivalent before and after LASIK

Degree of myopia	Baseline	3-day followup	1-month followup	6-month followup	1-year followup
Mild	-2.07 ± 0.32	+0.39 ± 0.10	+0.12 ± 0.04	-0.12 ± 0.03	-0.09 ± 0.03
Moderate	-4.93 ± 0.69	+0.62 ± 0.34	+0.24 ± 0.21	-0.17 ± 0.09	-0.33 ± 0.27
Severe	-7.95 ± 1.74	+0.66 ± 0.40	-0.53 ± 0.23	-1.77 ± 0.38	-1.88 ± 0.36

The refractive results for LASIK are presented in table 1. The paired t-test was used to compare the mean SEQ before and after LASIK for each degree of myopia in the two subgroups. Compared to the baseline, the followup results at each postoperative time point demonstrated statistically significant changes (p<0.05)(Table 1).

At the 1-year followup, the mean myopic regression in IntraLASIK subgroup 1 was 0.56 ± 0.18 D (p≤0.01). In the LASIK group, 70% of the eyes examined were within ± 0.5 D of the refractive target, and 85% of them were within ±1 D of the refractive target. In IntraLASIK subgroup 2, the mean myopic regression was 1.4 ± 0.3 D (p≤0.01). Approximately 90% of the eyes examined were within ±0.5 D of the refractive target, and 94% of the eyes examined were within ±1 D of the refractive target.

There was no difference in visual acuity between the LASIK and IntraLASIK groups. Approximately 18% and 13% of the patients in the IntraLASIK and LASIK groups, respectively, achieved better vision by gaining 1 or 2 lines after correction. At 6-month and 12-year followup, there was no significant difference in the UCVA between the LASIK and IntraLASIK groups (p≥0.05).

Patients with high ametropia responded significantly differently to the two treatments (Table 2).

**Table 2:** Mean uncorrected visual acuity before and after IntraLASIK and LASIK

Surgery type	Degrees of myopia	Baseline	3-day followup	1-month followup	6-month followup	1-year followup
IntraLASIK	Mild	0.13 ± 0.05	0.97 ± 0.05	0.99 ± 0.01	0.99 ± 0.01	0.99 ± 0.01
IntraLASIK	Moderate	0.08 ± 0.05	0.81 ± 0.02	0.97 ± 0.02	0.99 ± 0.01	0.99 ± 0.01
IntraLASIK	Severe	0.04 ± 0.02	0.43 ± 0.09	0.49 ± 0.04	0.46 ± 0.16	0.49 ± 0.06
LASIK	Mild	0.11 ± 0.06	0.94 ± 0.07	0.97 ± 0.03	0.97 ± 0.03	0.97 ± 0.02
LASIK	Moderate	0.08 ± 0.07	0.92 ± 0.05	0.96 ± 0.03	0.99 ± 0.02	0.97 ± 0.03
LASIK	Severe	0.04 ± 0.01	0.42 ± 0.04	0.41 ± 0.02	0.34 ± 0.08	0.33 ± 0.04

Compared to the baseline, the UCVA in the IntraLASIK group significantly improved at all followup time points (p<0.05, Table 2). Compared to the baseline, the UCVA in the LASIK group significantly improved at all followup time points (p<0.05, Table 2).

The tissue-preserving surgery was more effective in maintaining visual acuity than standard interventions, especially in patients with high ametropia.

The standard ablative intervention, unlike LASIK or other standard procedures, does not require reducing the optical zone, as this may lead to a decrease in stromal thickness and worsen vision. Postoperatively, a decrease in CH and CRF was observed in both study groups (p≤0.05). The amount of reduction varied according to the flap creation technique and the level of laser exposure. The mean decrease in CH and CRF averaged 37% from the baseline in the LASIK group and 27% from the baseline in the IntraLASIK group (Tables 4 and 5). At the 6-month followup, a similar outcome was seen (37% vs. 28% reduction in the LASIK vs. IntraLASIK group). As laser exposure increased, the reduction effect increased (the level of laser exposure depended on the myopic severity) in the LASIK and IntraLASIK groups.

**Table 4:** Predicted stromal thickness after tissue-preserving and standard ablation, taking myopia severity into consideration

Myopia, D	Optical zone diameter, mm	Stromal thickness in tissue-preserving ablation, µm	Stromal thickness in standard ablation, µm
-10.0	5.8	247.9	219.1
-8.75	5.8	250.9	221.1
-8.50	5.9	251.1	220.1
-8.25	6.0	251.1	219.1
-8.15	6.1	250.1	215.6
-7.90	6.2	260.0	216.5
-7.65	6.3	251.1	214.6
-7.40	6.4	251.8	215.1
-7.25	6.5	250.5	213.9

The parameters measured were as follows: central corneal thickness, which was 470 µm; and keratometry, which measured 43 D.

**Table 5:** Predicted stromal thickness after tissue-preserving and standard ablation, taking myopia severity into consideration

Myopia, D	Optical zone diameter, mm	Stromal thickness in tissue-preserving ablation, µm	Stromal thickness in standard ablation, µm
-10.0	5.8	331.3	330.0
-9.90	5.9	330.0	329.8
-9.60	6.0	331.1	330.3
-9.25	6.1	331.6	330.7
-8.90	6.2	330.9	330.1
-8.50	6.3	334.6	333.8
-8.25	6.4	334.6	333.7
-8.00	6.5	334.6	333.9

The parameters measured were as follows: central corneal thickness, which was 520 µm; and keratometry, which measured 43 D.

## DISCUSSION

Refractive surgery in myopic patients with thin corneas (<520 µm) is significantly challenging^(10)^. LASIK, a popular option, improves visual acuity and rarely causes complications^(9^,^11)^. However, corneal thickness reduction and biomechanical changes, particularly in thin corneas, remain a concern^(12)^. Furthermore, LASIK may be associated with complications, such as iatrogenic corneal keratectasia and small flaps, which often occur due to inadequate residual stromal bed thickness^(8^,^13)^. The uncertainty of mechanical microkeratomes further contributes to complications^(7)^. In contrast, Femto-LASIK allows more precise flap cutting and customizable flap sizes, which addresses these concerns with LASIK^(14)^.

For excimer laser ablation, patients with severe myopia and thin corneas represent a challenging group. The main difficulties associated with excimer laser ablation are achievement of target refraction and maintenance of desired vision. Furthermore, the stromal thickness should be >250 µm. Excimer laser correction techniques for these risk groups have been optimized^(15^,^16)^. These advances allow ophthalmologists to handle high diopters (D) and perform surgical interventions when the corneal thickness is insufficient.

**Table 3:** Predicted stromal thickness after tissue-preserving and standard ablation

Optical zone diameter, mm	Stromal thickness after tissue-preserving ablation, µm	Stromal thickness after standard ablation, µm
5.8	287.8	253.8
5.9	284.7	248.7
6.0	281.7	248.7
6.1	278.3	238.7
6.2	274.1	232.5
6.3	271.6	226.7
6.4	268.2	220.7
6.5	264.8	214.9

The parameters measured were as follows: central corneal thickness, which was 520 µm; keratometry, which measured 43 D; and myopia, which was 10 D.

The effectiveness of keratorefractive surgery can be assessed by measuring refractive indices and visual acuity before and after the intervention. The long-term stability of the achieved result is also crucial. The most commonly used tests for examination are keratometry and pachymetry, and comprehensive assessments can also include corneal topography. However, findings obtained with these techniques may not be sufficient for diagnosing keratoconus^(17)^. This is especially true for subclinical keratoconus and patients with reduced corneal thickness. Thus, more studies are required to more accurately predict intraoperative and postoperative complications of keratorefractive surgery. Corneal biomechanics also remain insufficiently studied, especially the postoperative dynamics. The common methods used to study the structural parameters of the cornea include the microscopic examination of the posterior epithelium, confocal microscopy, and optical coherence tomography^(18)^. The biomechanical properties of the cornea can be assessed with an ocular response analyzer (ORA)^(19)^.

Our study compared the IntraLASIK and LASIK procedures for myopia correction in patients with different corneal characteristics. Although both surgeries significantly improved visual parameters, there were notable differences. LASIK achieved greater SEQ correction initially than IntraLASIK did.

This may be attributable to LASIK’s more intense laser impact and thinner corneal flap. However, IntraLASIK demonstrated a stable and effective long-term correction. Both methods yielded a high postoperative visual acuity, with similar outcomes over time. The complication rates between the two methods did not significantly differ. However, both procedures may affect the corneal biomechanics, which may necessitate close postoperative monitoring. Overall, both IntraLASIK and LASIK are safe and effective options for myopia correction, and the choice of procedure depends on individual patient factors.

Myopia is a very common disorder that affects a significant portion of the population worldwide. Therefore, the effective correction of myopia remains crucial in current studies. The number of laser eye surgeries has been steadily increasing over the past decade. Some studies have reported the rise in FemtoLASIK in different countries around the world, including China^(20^,^21^,^22)^. However, more studies on excimer laser surgery are required to ensure effective treatment.

Some studies state that FemtoLASIK and LASIK have similar postoperative complications^(21)^, including diffuse lamellar keratitis, dry eye, light sensitivity, flap dislocation, epithelial ingrowth, and keratectasia^(22).^ However, none of these adverse events were observed in our study. Thus, the risk of developing complications after LASIK and IntraLASIK remains debatable.

Studies continue to assess the creation of corneal flaps of the required size and monitor the postoperative corneal biomechanics^(23)^. Our study findings demonstrate that IntraLASIK provides a better outcome at the 1-year follow-up than LASIK. Furthermore, IntraLASIK allows the creation of a corneal flap of the desired size with a higher accuracy than LASIK, even in patients with thin corneas. However, when constructing a flap, surgeons alter the anatomical properties of the cornea and may compromise the innervation, causing changes in the corneal biomechanics.

According to some authors, laser ablation can induce biomechanical changes at the center as well as the periphery of the cornea^(24)^, leading to an increase in refractive properties. Other authors have reported a decrease in CH and CRF after FemtoLASIK as a general corneal response to laser therapy^(25)^. In the current study, IntraLASIK and LASIK had the same effect on the corneal biomechanical properties. The redaction rate was significantly lower with IntraLASIK than with LASIK. In this study, the biomechanical behavior of the cornea was affected by the amount of laser exposure, myopic severity, and corneal thickness. This finding is consistent with findings from other studies^(26^,^27)^.

The postoperative changes and complications in the cornea are detectable via confocal microscopy. Dry eyes are associated with a decrease in stromal keratocytes due to cellular apoptosis in the ocular surface tissue. Another serious complication of laser treatments is keratectasia, which can occur postoperatively in the presence of thin corneas, severe myopia, subclinical keratoconus, and anterior radial keratotomy and when the corneal curvature is ^3^ 44 D^(28^,^29^,^30)^. In the present study, we assessed the effect of a thin cornea and severe myopia on visual outcomes.

Another tissue-preserving technology, sub-Bowman’s keratomileusis, enables the adjustment of an additional diopter. However, it is ineffective in patients with severe myopia and a corneal thicknesses of <460 ×m^(31)^. The intraocular procedure for the correction of high myopia, such as IOL implantation, is underdeveloped and does not always produce the intended functional results.

The deviation from the targeted outcome is a significant issue in laser treatments^(32)^. Although IntraLASIK was more accurate than LASIK in this study, the issue persisted. This deviation has been attributed to errors in morphometry, displacement of the intraocular lens, and postoperative alterations in refractive power^(27^,^32)^. Myopia is more common in younger people with the ability to accommodate than in older people. However, intraocular lenses can reduce the accommodative reserve of the eye^(28)^. In this study, IntraLASIK was more effective than LASIK in maintaining the corneal biomechanical changes and visual acuity throughout the study period.

The study findings highlight the efficacy of IntraLASIK in achieving precise corneal flap creation across varying corneal thicknesses with minimal deviation from the intended parameters. IntraLASIK’s ability to create uniform flaps reduces the risk of deviations and ensures accurate refractive correction, which is particularly advantageous for patients with significant myopia and thin corneas. Myopic regression is significantly lesser after IntraLASIK than after LASIK, reinforcing its preference in such patients. The study’s limitations include a limited sample size and short observation period. Further studies are required to explore the long-term outcomes of laser treatments such as LASIK and IntraLASIK, and to compare various correction methods.

Further studies are required to explore biomechanical changes and long-term outcomes to refine treatment selection.

Furthermore, investigating the impact of factors such as age and comorbidities on surgical effectiveness and safety could provide further insights into the outcomes of surgical procedures. Moreover, assessing the influence of technical parameters such as flap dimensions and treatment zones on visual correction outcomes and corneal biomechanical characteristics could enhance understanding and refinement of treatment strategies. The study findings offer valuable guidance for clinicians and patients in selecting optimal myopia correction methods according to individual characteristics. Furthermore, it emphasizes the importance of ongoing studies to refine treatment approaches and improve patient outcomes.

Our study aimed to compare the efficacy and safety of LASIK and IntraLASIK in addressing myopia. The results indicate that IntraLASIK produced more precise flaps than LASIK, with deviations of <8 µm and 0.1 mm from the intended thickness and diameter, respectively. LASIK resulted in nonuniform flaps, with thickness deviations of 5-86 µm. Additionally, IntraLASIK demonstrated superior efficacy for patients with severe myopia and thin corneas, achieving a mean spherical equivalent of 0.9 D at 6 months compared to 1.4 D for LASIK. Furthermore, approximately 91% and 83% of patients with mild to moderate and severe myopia, respectively, achieved results within ±0.49 D from the refractive target with IntraLASIK.

Our findings suggest that corneal hysteresis and corneal resistance factor decrease with an increase in laser intensity, and they decrease faster with thinner corneas. Therefore, IntraLASIK may be more beneficial than LASIK for patients with thin corneas and severe myopia.

### Clinical Implications

The superior efficacy and precision of IntraLASIK, particularly for patients with thin corneas and severe myopia, have significant implications for clinical practice. Ophthalmologists should consider these findings when selecting the most suitable laser-assisted intervention for myopia correction, aiming to achieve optimal visual outcomes while minimizing potential risks.
